# Characterization of intrauterine growth, proliferation and biomechanical properties of the murine larynx

**DOI:** 10.1371/journal.pone.0245073

**Published:** 2021-01-13

**Authors:** Kate Griffin, Hailey Pedersen, Kari Stauss, Vlasta Lungova, Susan L. Thibeault

**Affiliations:** 1 Division of Otolaryngology, Department of Surgery, University of Wisconsin-Madison, Madison, Wisconsin, United States of America; 2 Department of Biomedical Engineering, University of Wisconsin-Madison, Madison, Wisconsin, United States of America; University of Colorado School of Medicine, UNITED STATES

## Abstract

Current research approaches employ traditional tissue engineering strategies to promote vocal fold (VF) tissue regeneration, whereas recent novel advances seek to use principles of developmental biology to guide tissue generation by mimicking native developmental cues, causing tissue or allogenic/autologous progenitor cells to undergo the regeneration process. To address the paucity of data to direct VF differentiation and subsequent new tissue formation, we characterize structure-proliferation relationships and tissue elastic moduli over embryonic development using a murine model. Growth, cell proliferation, and tissue biomechanics were taken at E13.5, E15.5, E16.5, E18.5, P0, and adult time points. Quadratic growth patterns were found in larynx length, maximum transverse diameter, outer dorsoventral diameter, and VF thickness; internal VF length was found to mature linearly. Cell proliferation measured with EdU in the coronal and transverse planes of the VFs was found to decrease with increasing age. Exploiting atomic force microscopy, we measured significant differences in tissue stiffness across all time points except between E13.5 and E15.5. Taken together, our results indicate that as the VF mature and develop quadratically, there is a concomitant tissue stiffness increase. Greater gains in biomechanical stiffness at later prenatal stages, correlated with reduced cell proliferation, suggest that extracellular matrix deposition may be responsible for VF thickening and increased biomechanical function, and that the onset of biomechanical loading (breathing) may also contribute to increased stiffness. These data provide a profile of VF biomechanical and growth properties that can guide the development of biomechanically-relevant scaffolds and progenitor cell differentiation for VF tissue regeneration.

## Introduction

The vocal folds (VF), seated in the larynx, are the primary tissue involved in phonation. VF scarring, which occurs after injury to the VFs, is the greatest cause of voice disorder, and remains one of the VF conditions most resistant to treatment [1, 2]. Individuals with vocal impairments and dysphonia have nearly as many short-term disability claims and work productivity losses as people suffering from chronic conditions such as depression, asthma, and heart disease [3]. VF scarring is associated with extracellular matrix (ECM) fibrosis in the lamina propria, which directly alters phonation and vibratory function. Treatment outcomes for VF scarring and ECM injury or loss remain largely unsuccessful, despite current therapeutic efforts [1].

While there is promising interest in using tissue engineering approaches to replace or heal injured VF tissue, substantial limitations in cell sourcing exist [4]. Primary cell sources are nearly impossible to obtain, as their collection is highly invasive and harmful to native tissue. Moreover, current differentiation factors for stem and progenitor cells are associated with the stimulation of fibrotic phenotypes (abnormal biomechanical properties, matrix content and organization), which limits their current usage and is not conducive to the mimicry of native, healthy tissue [5–8]. To circumvent these issues, a more recent tissue engineering approach seeks to provide stem and progenitor cells with embryogenic biological and biomechanical signals to drive differentiation and gross organization [9, 10]. Cellular biomechanical environmental cues, including tensile loading and elastic modulus changes, have been shown to potently mediate differentiation [11–14]. Fibroblasts and stem cells have exhibited modified behaviors in response to biomechanical stimuli, and muscle-driven movements beginning at E12.5 in mice and week 10 in humans, suggesting that both static and dynamic stimulation, including matrix stiffness and stretch, occurs during differentiation [15].

Mice are an established model to study laryngeal development in humans; their similar laryngeal framework and distribution of elastin and collagen fibers in the lamina propria makes them a good model for biomechanical studies, and their relatively quick and well-defined gestation period makes them well-suited to genetic studies [16]. Embryonic morphological VF development in mice has been previously defined, but many important functional properties remain uncharacterized [17–19]. There is a significant paucity in the literature for soluble factors and biomechanical data specific to VF development from initiation through stratification into a functional, voice-producing organ. Biomechanical forces are expected to change as the VFs proliferate and grow, providing dynamic cues to progenitor cell differentiation.

Bulk biomechanical properties are commonly tested using uniaxial tensile testing, which is best suited to larger, stronger tissues than the VFs. Atomic force microscopy (AFM) based indentation is an established method to characterize elasticity of small, fragile biological tissues [20] and has previously been used to characterize embryonic chick tendon biomechanical developmental profiles, the morphology and elasticity of lamina propria collagen fibrils, and the gross elasticity, adhesion, and surface roughness of the VF lamina propria [20–22]. Depending on probe tip size, AFM can be used to measure different scales (e.g. nano, micro) of tissue elastic modulus.

The identification and combination of embryonic biomechanical and chemical cues will allow researchers to precisely mimic the embryonic developmental environment. This knowledge will inspire the creation of developmentally-inspired scaffolds for injured tissue regeneration, optimize and expand cell sources with the goal of advancing developmental biology-inspired tissue engineering for the VFs. Embryogenically-informed cell differentiation programs have already met with success in tendon, cartilage, and muscle tissues [11–13]. Here, we seek to lay groundwork for embryogenic differential cues through characterization of murine intrauterine growth, proliferation, and elastic modulus at key time points in murine VF development.

## Materials and methods

### Mouse mating and tissue collection

Using wild-type FVB/N mice, males and females were mated and pregnant female mice were sacrificed at embryonic (E) days E13.5, E15.5, E16.5, E18.5, postnatal (P) stages, P0, and adult (6–8 weeks). To determine embryonic age, noon of the day when the vaginal plug of the pregnant mouse was found was labeled as E0.5. Sacrifices were performed following regulations of protocols approved by the University of Wisconsin Animal Care and Use Committee. Briefly, adult mice were euthanized using CO_2_. Anesthesia in neonatal mice was induced by hypothermia followed by decapitation. Twenty mice per time point were collected for VF measurements and cell proliferation; three to four mice per time point were used for biomechanical data. For growth and proliferation measurements, whole embryos were dissected for E13.5-E16.5, the neck region was dissected for E16.5-P0, and the whole larynx was dissected for adult mice. All embryonic samples were immediately fixed in 4% paraformaldehyde (PFA) in phosphor-buffered saline (PBS) overnight, whereas all adult samples were immediately fixed in 10% formalin overnight. These samples were then embedded in paraffin blocks for sectioning in 5 μm serial sections. Coronal sections for vocal fold thickness were performed on 10 blocks, and transversal sections for vocal fold length were collected from the remaining 10. Each section was rehydrated for hematoxylin and eosin staining, immunofluorescence (IF) staining for SOX9 and Desmin (E13.5 only), and EdU assay. Biomechanical study samples were completed on fresh tissue, which were embedded in low-melt agarose and sectioned using a vibratome in 150 μm transverse sections.

### Histology and immunofluorescence (IF)

Hematoxylin and eosin (H&E) staining was performed on 10 coronal and 10 transverse sections for VF measurements of thickness and length. Samples were deparaffinized and dehydrated, placed into series of chemicals (hematoxylin, acid ethanol, ammonia, and eosin), dehydrated, and mounted in cytoseal. Sections were used for vocal fold thickness and length measurements. Double IF stains for SOX9 (expressed in cartilage and cartilage precursors) and Desmin (skeletal muscle marker) were performed on 10 coronal and 10 transverse E13.5 sections to determine thyroid and cricoid cartilage and thyroarytenoid muscle locations when not visible under H&E. All other time points were stained for only H&E. Samples were deparaffinized and rehydrated, then blocked in 10% bovine serum albumin (BSA) in PBS. SOX9, diluted 1:300 (EMD Millipore; Billerica, MA) and Desmin, diluted 1:100 (Sigma Aldrich; St. Louis, MO) were added and incubated at 4° C overnight. Following PBS + Tween washes, anti-rabbit secondary antibody, diluted 1:800, was applied for 35 minutes. This process was then repeated with antimouse secondary antibody diluted 1:800. Following a final was series in PBS + Tween, slides were stained with 1:10,000 DAPI (Molecular Probes, Eugene, OR) for 1 minute prior to mounting with fluoromount.

### EdU assay

Adult and pregnant female mice received an intraperitoneal injection of 5’-ethynyl-2’-deoxyuridine (EdU) an hour prior to sacrifice. Six samples (three coronal, three transverse) were deparaffinized, rehydrated, and solubilized using xylene, an ethanol series, and 0.5% Triton respectively. Following the Click-iT® EdU Alexa Fluor® 488 Imaging Kit protocol, reaction buffer was prepared using Click-iT® reaction buffer, Copper (II) Sulfate (CuSO_4_), Alexa Flour® azide, and reaction buffer additive. Samples were coated and incubated for 30 minutes in dark, humidified chamber and room temperature. After PBS + Tween washes, DAPI (1:10,000) was added for 1 minute prior to mounting with fluromount.

### Laryngeal and VF measurements

**Fig 1:**
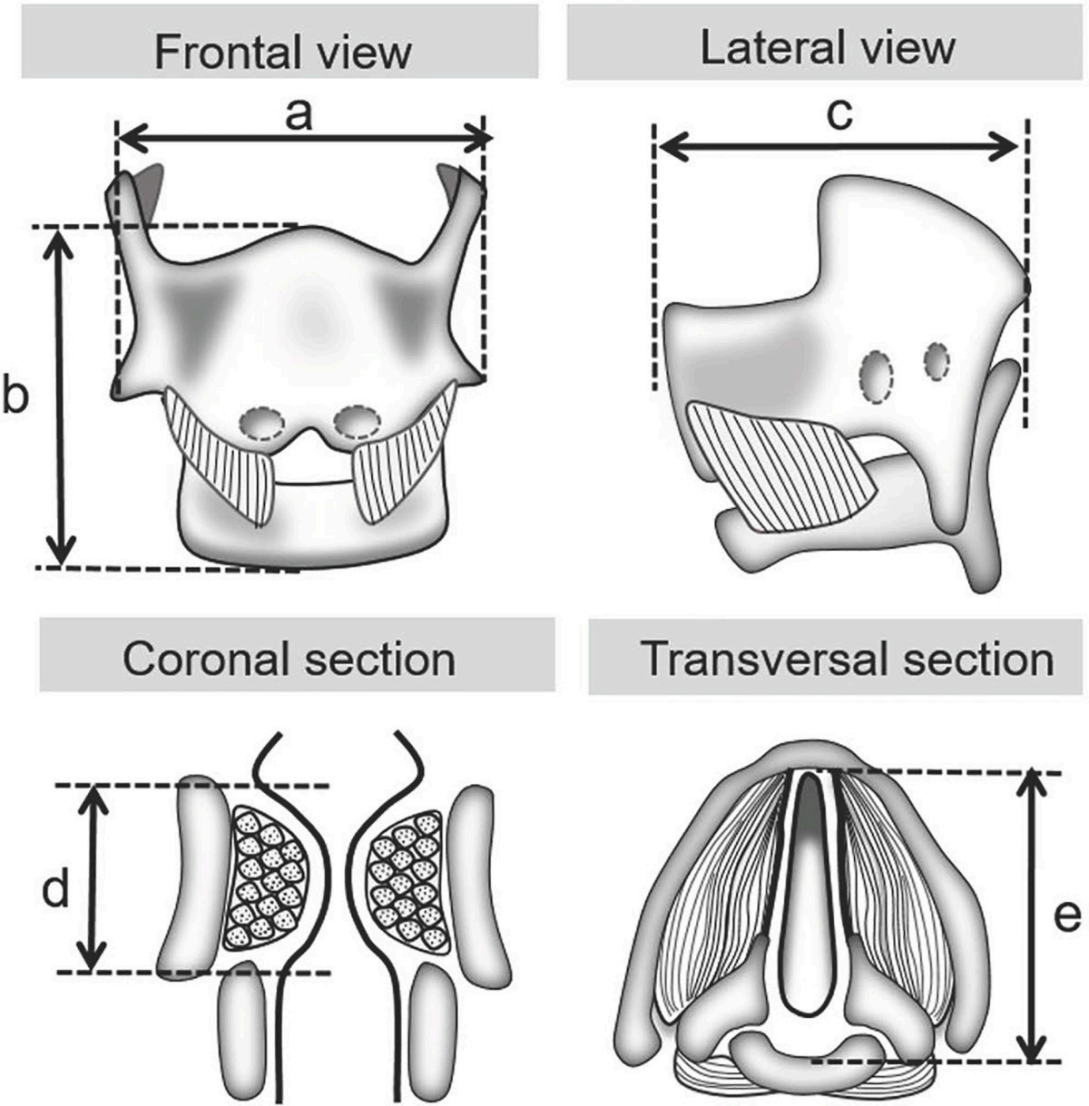
Schematic of larynx and vocal folds for growth measurements. (a) Laryngeal transverse diameter. (b) Laryngeal length. (c) Laryngeal outer dorsoventral diameter (d) Vocal fold thickness. (e) Vocal fold internal length.

Images were taken at 4 to 20x, dependent on stage, in order to have the entire laryngeal region (from thyroid to cricoid) visible for measurements. Laryngeal images were taken using a Zeiss stereomicroscope attached to a Canon T4i 18 MP digital camera and uploaded into ImageJ, which was calibrated to provide scale. VF images were taken using a Nikon Eclipse attached to an Olympus digital camera and uploaded into ImageJ, which was calibrated to provide scale for all images with the exception of EdU stained samples. Laryngeal measurements were taken from H&E stained images as follows (with double IF stains on E13.5 samples): maximum transverse diameter–measured across the front of the thyroid shield (Fig 1A), length of the larynx–measured from the epiglottis to the inferior margin of the cricoid cartilage midline (Fig 1B), outer dorsoventral diameter–measured from the thyroid anteriorly to the cricoid posteriorly (Fig 1C). Vocal fold measurements of thickness–measured from superior epithelium directly above the thyroarytenoid muscle (TA muscle) to the inferior margin of the TA muscle (Fig 1D) and internal length–from the thyroid to the cricoid (Fig 1E) were taken. E13.5 samples did not have differentiated TA muscle within the larynx; VF thickness measurements were performed from superior epithelium to cricoid cartilage for continuity. Cellular proliferation measurements were taken using EdU stained slides. EdU positive cells were counted at larynx level excluding cartilage cells. For laryngeal, vocal fold, and proliferation measurements both inter- and intra-rater reliability was calculated for each stage; 10% of measurements were repeated for comparison.

### FV-AFM

**Fig 2:**
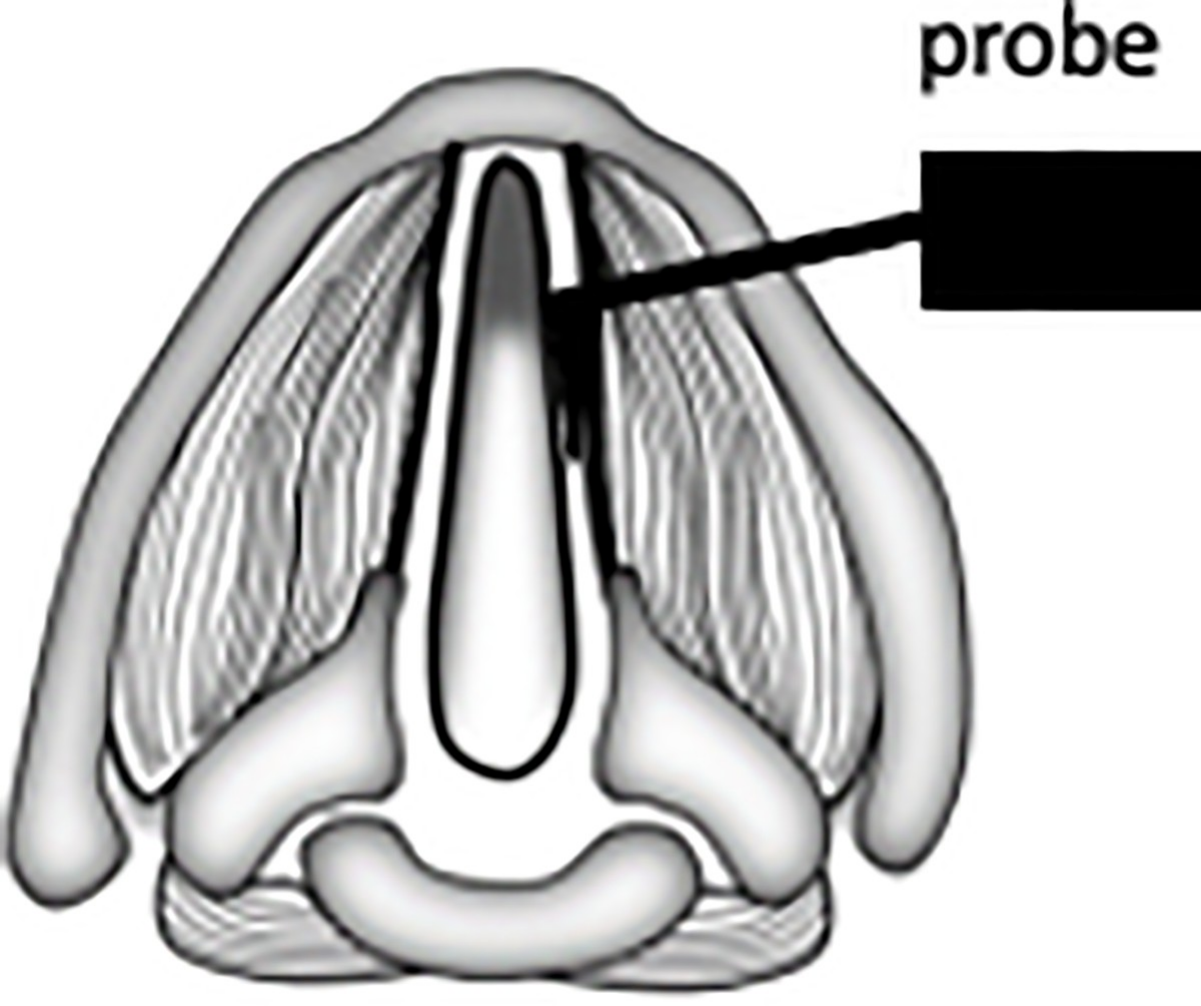
Schematic of AFM sampling for biomechanical stiffness. Probe is placed on lamina propria and repeated indentation measurements are taken.

Larynges collected for biomechanical characterization were embedded in 4% w/v low-melt agarose (Dot Scientific, Burton, MI) and sectioned in 150 μm transverse sections using a vibratome set at a frequency of 8. Samples were placed on adhesive backed glass slides but remained unfixed. Force curves were acquired on the Bruker Catalyst Bio-AFM (Bruker Nano Surfaces, Santa Barbara, CA) using silicon nitride cantilevers (Novascan Technologies, Boone, IA) with spherical tip (r = 5 μm), nominal spring constant = 0.06 N/m, and tip half angle of 18°. The cantilever was held using the commercial fluid tip holder and were calibrated before each experiment with a deflection sensitivity measurement on glass in fluid and a simple harmonic oscillator thermal tune. Single indentation force curves for each time point were taken on three samples at three spots per sample, with 20 readings per spot (180 force curves) (Fig 2). Probes descended 8 μm into tissue, indenting 5.3% of tissue thickness, which limited contributions from the underlying glass slide (indentation depth must be less than 10% of overall tissue thickness). Tissues were kept submerged in PBS to keep them moist and force curve readings were acquired in fluid at room temperature.

Slopes of the linear regions of the curves were calculated theoretically using the Hertzian indentation model using a sample Poisson’s ratio of 0.300. Using the Nanoscope Analysis 1.5 Software (Bruker Nano Surfaces, Santa Barbara, CA), data from each set of samplings was viewed and a representative curve selected. Baseline corrections were performed on the force curve, and elastic modulus was measured from the slope of the curve. The representative curve analysis was then used as the template for a pipeline data analysis by the Nanoscope software.

Data from the pipeline analysis of each sample spot was pooled into data for each time point. AFM reading error reported a Young’s modulus of -1.000; these data points were filtered out from the data. Histograms were plotted to view data distributions. Outliers were identified and removed using the 1.5 x interquartile range (IQR) filter, which removes outliers based on median values rather than means.

### Statistical analysis

For all data, means and standard deviations were calculated. ANOVA and a post-hoc Tukey test were used for growth measurements; data normality and homogeneity of variance were validated via QQ-plots and Levene’s test, respectively. Inter- and intra-rater reliability was calculated via intraclass correlation coefficient with absolute agreement using a two-way mixed model, and was calculated for each embryonic stage of laryngeal, vocal fold, and proliferation measurements.

In order to determine data trends, linear, quadratic, cubic, and logarithmic models were fitted to each variable (larynx dorsoventral diameter, larynx length, larynx transverse diameter, VF internal length, VF thickness, transverse plane proliferation, coronal plane proliferation, and stiffness) across true time points (days since conception). Linear models were compared using ANOVA, and the best fit linear model was compared to the logarithmic model using Akaike’s Information Criteria (AIC), which allows comparison of linear and non-linear models. AFM measurements were tested for homogeneity of variance using the Fligner-Killeen test, which revealed heteroscedasticity in the data, which violates base assumptions for most statistical tests. Welch’s ANOVA and a post-hoc Games-Howell test, which do not require homogeneity of variance as a base assumption, were used to calculate significance. A p-value < 0.05 was considered statistically significant.

## Results

### E13.5 VF measurements influenced by thyroarytenoid muscle differentiation

Upon reviewing E13.5 IF stains, the thyroarytenoid muscle was not differentiated from other muscles within the larynx at this stage; VF thickness measurements were performed from superior epithelium to cricoid cartilage for measurement continuity. Analysis comparing H&E staining to IF staining of E13.5 revealed no statistically significant differences for internal length of the vocal folds (p = 0.5973); however, there was a significant difference for thickness of the vocal folds (p = 0.0061). This significant difference suggests that H&E staining alone is not sufficient for measurements in the coronal plane at this stage; SOX9 stains were used for E13.5 measurements [23, 24].

### Larynx and VF have quadratic growth profile

**Fig 3:**
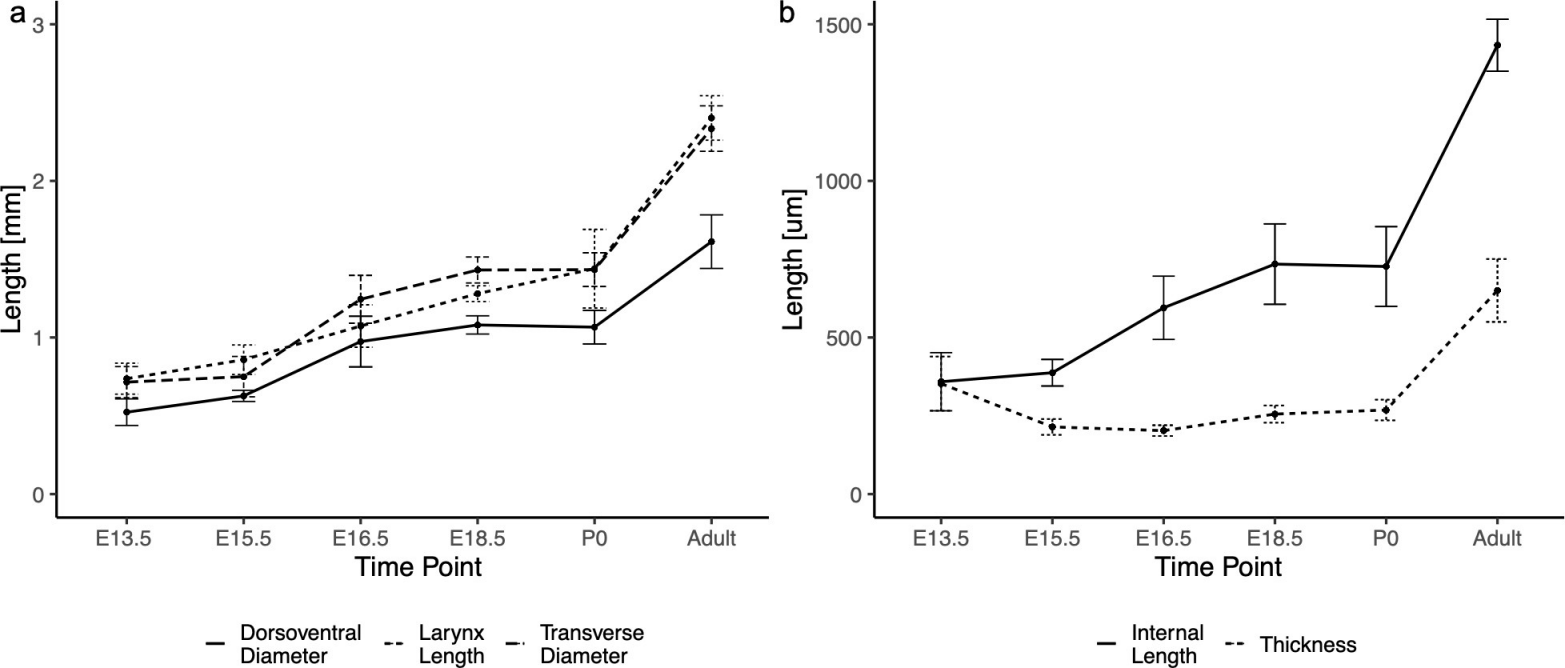
Growth measurements for larynx and vocal folds. (a) Graph of larynx measurements in dorsoventral diameter, larynx length, and transverse diameter. Quadratic growth patterns were found for all measurements. Comparisons of larynx length time points report statistically significant changes between all sequential time points except E13.5-E15.5, E16.5-E18.5, and E18.5-P0. Outer dorsoventral diameter was significant between all sequential time points except E13.5-E15.5 and E18.5-P0. Transverse diameter was significant between all sequential time points except E13.5-E15.5 and E18.5-P0. (b) Graph of measurements of the vocal fold in internal length and thickness, shown by time point. Internal VF length increases linearly while VF thickness increases quadratically. Comparisons of VF measurement time points reported significant changes between all internal length time points except E13.5-E15.5 and E18.5-P0. Thickness was significant between all sequential time points except E15.5-E16.5 and E18.5-P0.

H&E staining was performed to identify the exact placement of the VFs in morphological assessment. IF stains for SOX9, expressed in cartilage and cartilage precursor cells, and Desmin, a skeletal muscle marker, helped identify the VFs when the thyroid and cricoid cartilages and thyroarytenoid muscle have not fully formed (E13.5). Mean larynx lengths were measured as E13.5 0.74 ± 0.1 cm, E15.5 0.86 ± 0.09 cm, E16.5 1.07 ± 0.14 cm, E18.5 1.28 ± 0.05 cm, P0 1.44 ± 0.25 cm, adult 2.40 ± 0.14 cm. Mean transverse diameter were measured as E13.5 0.72 ± 0.1 cm, E15.5 0.75 ± 0.13 cm, E16.5 1.24 ± 0.15 cm, E18.5 1.43 ± 0.08 cm, P0 1.43 ± 0.11 cm, adult 2.33 ± 0.15 cm. Mean outer dorsoventral diameter were measured as E13.5 0.52 ± 0.08 cm, E15.5 0.63 ± 0.04 cm, E16.5 0.97 ± 0.16 cm, E18.5 1.08 ± 0.06 cm, P0 1.07 ± 0.12 cm, adult 1.61 ± 0.17 cm. Results are shown in Fig 3A (true time points in S1 Fig). Length of the larynx significantly (p<0.05) differed between E13.5 and E16.5/E18.5/P0/adult, E15.5 and E16.5/E18.5/P0/adult, and E16.5 and E18.5/P0/adult, E18.5 and adult, and P0 and adult (S2 Table). Similar results were found for maximum transverse diameter of the larynx and outer dorsoventral diameter of the larynx. Inter and intra-reliability ICC values are reported in Table 1.

**Table 1:** Inter- and intra-reliability for larynx and VF measurements.

Measurement	Inter-rater ICC	Intra-rater ICC
**Length**	0.988	1.000
**Maximum Transverse Diameter**	0.999	0.999
**Outer Dorsoventral Diameter**	0.999	0.997
**E13.5 Transverse**	0.988	0.998
**E13.5 Coronal**	0.998	0.998
**Internal Length**	0.999	1.000
**Thickness**	0.999	0.997
**Transverse EdU**	0.996	0.996
**Coronal EdU**	0.992	0.727

Inter- and intraclass correlation with absolute agreement, two-way mixed model.

Mean VF internal lengths were measured as E13.5 359 ± 93 μm, E15.5 387 ± 42 μm, E16.5 595 ± 101 μm, E18.5 734 ± 128 μm, P0 727 ± 127 μm, adult 1433 ± 83 μm. Mean vocal fold thicknesses were measured as E13.5 353 ± 87 μm, E15.5 214 ± 25 μm, E16.5 203 ± 17 μm, E18.5 255 ± 28 μm, P0 268 ± 33 μm, adult 650 ± 100 μm (Fig 3B, true time points in S1 Fig). Significant differences were measured for internal VF length between E13.5 and E15.5 and between E18.5 and P0 (S3 Table). Finally, a significant difference for vocal fold thickness was noted between all stages except E15.5 and E16.5/E18.5 and between E18.5 and P0. Inter and intra-reliability can be found in Table 1.

### Cellular proliferation decreases throughout development

**Fig 4:**
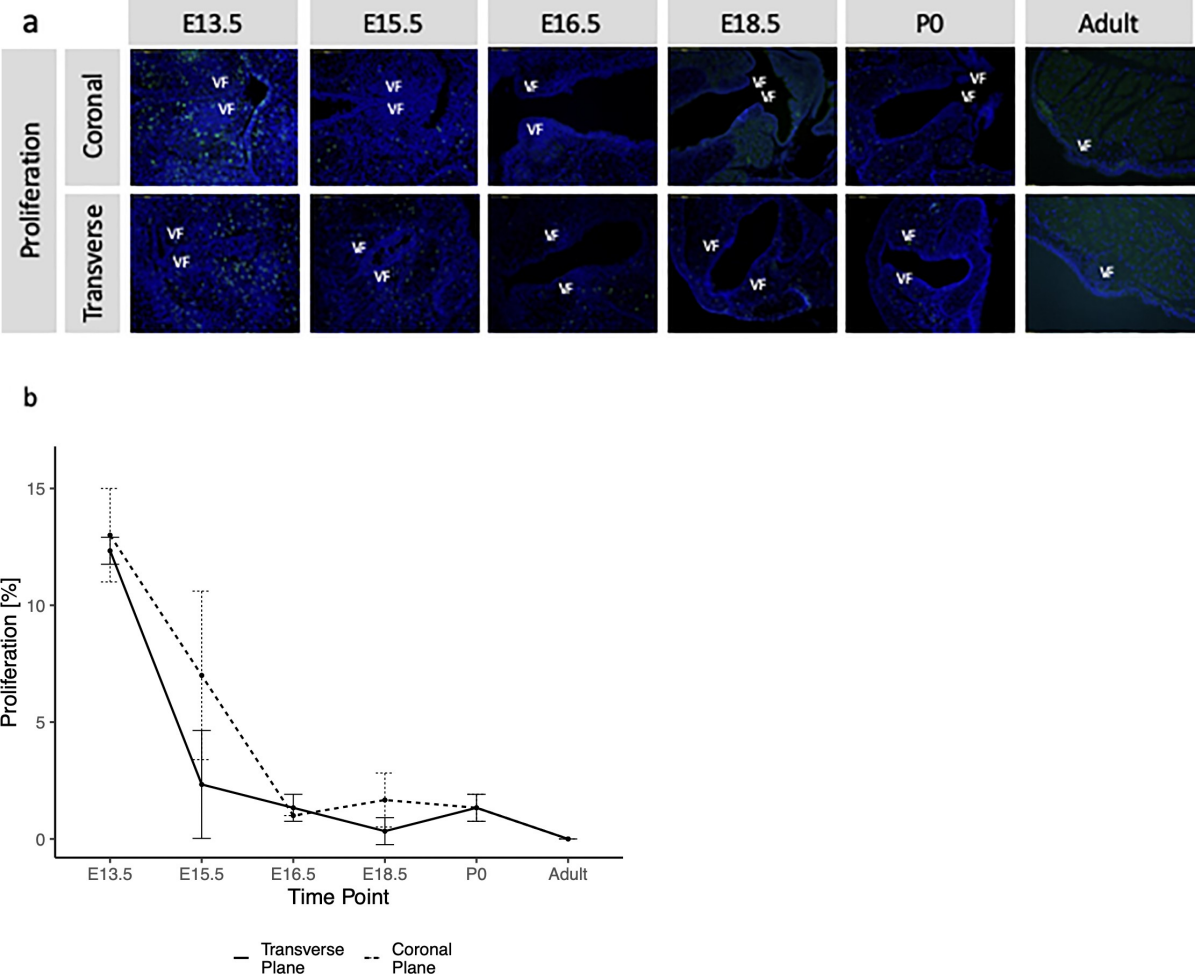
Proliferation measurements in transverse and coronal VF planes. (a) An EdU assay was performed to determine cellular proliferation in the laryngeal region. Coronal and transverse sections were taken at each time point. Cartilage cells were excluded from proliferation counts. Proliferation rates were significantly higher in early stages than at late stages. VF = vocal folds, bright green cells are those that are proliferating. (b) Graph of proliferation rates in the coronal and transverse VF, shown by time point. High decreases in proliferation are shown in early embryonic stages, and proliferation plateaus and stops by adult stages. Sequential comparison of coronal plane proliferation time points reported significant changes between E13.5-E15.5. Sequential comparison in the transverse plane reported significant differences between E13.5-E15.5 and E15.5-E16.5.

An EdU assay was performed to determine cellular proliferation in the laryngeal region (Fig 4A). EdU positive cells (cells that are bright green in Fig 4) were counted at the larynx level excluding cells within surround cartilages. Cellular proliferation in the transverse plane was measured (in %) as E13.5 13 ± 2, E15.5 7 ± 3.6, E16.5 1 ± 0, E18.5 1.7 ± 1.2, P0 1.3 ± 0.6, adult 0 ± 0. Cellular proliferation in the coronal plane was measured (in %) as E13.5 12.3 ± 0.6, E15.5 2.3 ± 2.3, E16.5 1.3 ± 0.6, E18.5 0.3 ± 0.6, P0 1.3 ± 0.6, adult 0 ± 0 (Fig 4B, true time points in S1 Fig). Significant differences (S4 Table) were measured in the transverse plane between E13.5 and E15.5/E16.5/E18.5/P0/adult, as well as between E15.5 and E16.5/P0/adult. Similar findings were noted for cell proliferation measured in the coronal plane; however, there were no significant differences between E15.5 and E16.5/P0/adult (S4 Table). Inter and intra-reliability are reported in Table 1.

Linear regression analysis found a significant quadratic (p = 0.049) best fit for coronal proliferation and a linear fit for transverse (p = 0.089, quadratic not significant) proliferation.

### VF tissue stiffness increases throughout development

**Fig 5:**
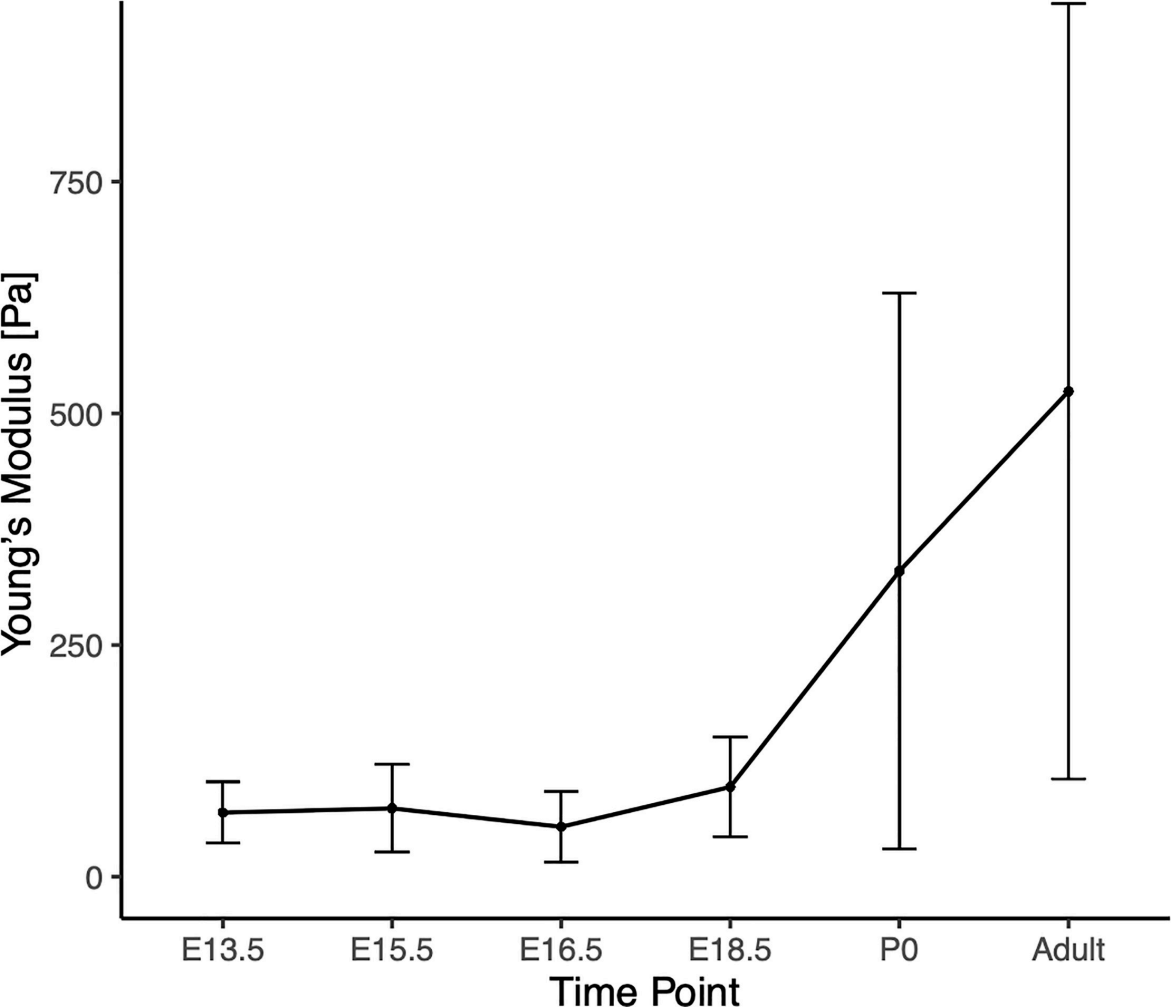
Graph of vocal fold stiffness, shown by time point. Stiffness was measured using atomic force microscopy. Statistical analysis found stiffness was found to increase quadratically over time. Large increases in variance evident with increase in time.

Using AFM contact mode in fluid, force curves were taken in triplicate samples at three spots per sample, with 20 readings per spot. Curves were analyzed using the Hertzian model. VF mean elastic moduli were measured as E13.5 69 ± 33 Pa, E15.5 74 ± 47 Pa, E16.5 54 ± 38 Pa, E18.5 97 ± 54 Pa, P0 329 ± 299 Pa, adult 523 ± 418 Pa (Fig 5). Statistically significant changes in modulus was measured between all time points except E13.5 and E15.5 (S4 Table). Linear regression analysis of VF stiffness found a best fit quartic model (p<0.001).

## Discussion

This study aimed to characterize growth, cell proliferation and biomechanics of the larynx and VFs using major anatomical landmarks, from intrauterine initiation of development to adulthood. Analysis of results revealed quadratic growth in larynx length, maximum transverse diameter, outer dorsoventral diameter, and VF internal length. Internal VF thickness was found to grow linearly, while proliferation decreases throughout development in a quadratic fashion in the coronal plane and linearly in the transverse plane; stiffness was found to quartic increase profile throughout development. These development profiles will be used to guide future development-driven studies for VF regeneration.

Overall, laryngeal growth was found to be quadratic. Lack of a statistical difference between E13.5 and E15.5 for the laryngeal growth measurements is supported by previous research which has demonstrated little to no proliferating cells at the site of vocal fold separation between E13.5 and E15.5, suggesting that structures of the larynx do not gain new cells, but rather that existing cells differentiate [17]. This is further supported by the decrease in cellular proliferation rates from E13.5 to E15.5 in both transverse and coronal planes (13% ± 0.02% vs 7% ± 0.04% and 12% ± 0.01% vs 2% ± 0.02% respectively). Lack of a significant difference for laryngeal growth measurements of length, maximum transverse diameter, and outer dorsoventral diameter between E18.5 and P0 can be explained by gestational dating as FVB/N mice typically give birth between 18 and 20 days. The mice used in this study were noted to give birth the same day that they were labeled to be E18.5, thus only a few hours often separated these stages.

VF stiffness increases quadratically throughout development. Notably, the variance in VF stiffness greatly increases as age increases; this same variance trend has been found in other embryonic stiffness developmental profiles such as chick tendon, likely due to increased variability in tissue maturation and stratification [21]. In early stages, the tissue is primarily composed of cells and disorganized ECM, while at later stages the ECM has begun to develop and stratify. The heterogeneity of the tissue surface may be responsible for the increase in variability at more developed stages. Further investigation is warranted.

Stiffness appears to increase gradually throughout development, rather than incrementally at key morphological stages, such as the onset (E13.5) and completion (E18.5) of recanalization of the VF. An initial decrease in stiffness at the beginning of recanalization, due to the loss of structural tissue support, would have been expected if stiffness was primarily influenced by morphology rather than tissue stratification, maturation, and ECM development. While morphological structure may contribute to overall tissue stiffness, it does not appear to be the primary driver of stiffness changes.

The large difference between pre- and post-natal stiffness profiles suggests that the changes between pre- and post-natal environments have a major effect on VF stiffness. It has been well documented that embryonic motility begins early, after neuromuscular connections form at E12.5 in the mouse, and 7 weeks in human embryos [15]. *In vivo* electromyography (EMG) recordings of sheep embryonic thyroarytenoid and posterior cricoarytenoid muscles show motor unit activation during fetal breathing and swallowing in the last two thirds of gestation [25, 26]. While EMG activity is not equivalent to force or strain, it suggests that the larynx and VF experience muscle derived forces during development [27]. Moreover, reduced or altered skeletal muscle contractions during embryonic development produce significant tissue abnormalities. While muscle contractions and swallowing motility begin *in utero*, these occur without associated atmospheric pressure changes [28]. In a normal breathing cycle, the lungs drop below atmospheric pressure during inspiration, and rise above atmospheric pressure during expiration. These pressure differentials occur not only in the lungs, but in the trachea, VFs, and larynx as well. The shift from muscle motility to cyclic pressure loading may play a role in the large VF stiffness increase between embryonic and postnatal stages, particularly between E18.5 and P0. The increase in stiffness may also be affected by the transition from fluid to air in the airway. Hydrated VF tissues are less stiff than dehydrated vocal tissues, an environmental factor which likely influences the stiffness changes in pre- and post-natal tissues [29]. While these environmental changes may be correlated to the indicated stiffness increase in postnatal stages, more studies are necessary to determine the exact cause for developmental increases in VF stiffness.

Significant negative trends for cellular proliferation in both the transverse and coronal plane suggests cellular proliferation slows as mice age, which corroborates findings in other embryonic studies [30, 31]. Decreasing proliferation rates during development indicate cellular proliferation is not responsible for concurrent increases in laryngeal growth, VF thickness, and VF stiffness. Increases in laryngeal and VF growth may be caused by extracellular matrix deposition, which would also contribute to VF stiffness increases, particularly during pre-natal development. Similar quadratic growth patterns in laryngeal and VF growth and VF stiffness indicates they may have shared contributing factors.

Collectively, results of this investigation suggest that signaling for VF progenitor cell differentiation will require dynamic changes in scaffold stiffness and proliferation signals. While this research gives important background and insight into VF development, more research needs to be conducted to determine what soluble factors may be necessary to combine with the developmental biomechanical profile.

## Conclusion

Murine VF growth, proliferation, and biomechanical development has been characterized through key developmental time points. Quadratic growth profiles are indicated in both the larynx and VF length, as well as the biomechanical stiffness of the VFs. Cellular proliferation has a significant negative trend, suggesting proliferation slows from initial stages of development to later stages, and that proliferation is not responsible for laryngeal growth or stiffness increases in the VFs. Post-natal stiffness gains suggest biomechanical loading may correspond to stiffness increases, rather than morphological VF growth. These results lay necessary background work for the pursuit of developmental biology-inspired tissue engineering strategies for the VF.

## Supporting information

S1 Fig Larynx growth measurements, vocal fold growth measurements, cellular proliferation measurements, and stiffness measurements graphed with true time points. (TIFF)

S1 Table P-value results for time point comparisons of larynx growth measurements. ANOVA reported significance between time points for larynx length (p<0.001), outer dorsoventral diameter (p<0.001), and transverse diameter (p<0.001). A post hoc Tukey test was conducted to report between time point significance.
(DOCX)

S2 Table P-value results for time point comparisons of VF growth measurements. ANOVA reported significance between time points for VF internal length (p<0.001) and thickness (p<0.001). A post hoc Tukey test was conducted to report between time point significance.
(DOCX)

S3 Table P-value results for time point comparisons of proliferation measurements. ANOVA reported significance between time points for coronal (p<0.001) and transverse (p<0.001) proliferation. A post hoc Tukey test was conducted to report between time point significance.
(DOCX)

S4 Table P-value results for time point comparisons of stiffness measurements. Welch’s ANOVA reported significance between time points for stiffness (p<0.001). A post hoc Games-Howell test was conducted to report between time point significance.
(DOCX)
