# Supplementary figures and images for: Characterization of intrauterine growth, proliferation and biomechanical properties of the murine larynx
Source: PLoS One. 2021 Jan 13;16(1):e0245073. doi: 10.1371/journal.pone.0245073 (PMC7806159; doi:10.1371/journal.pone.0245073)

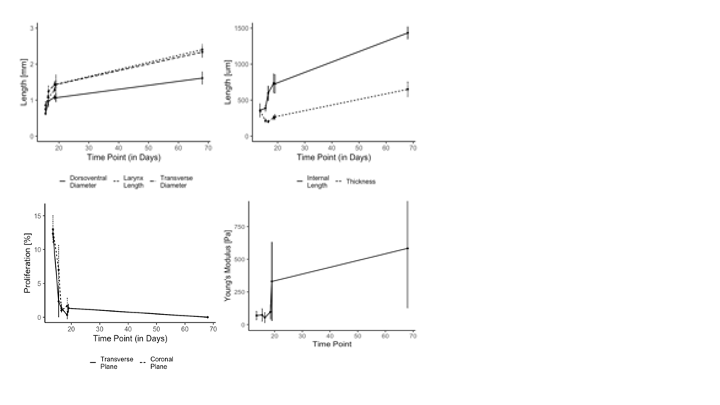

Supplement: S1 Fig — (TIFF) [file pone.0245073.s001.tiff]
