# Supplementary material for: Characterization of intrauterine growth, proliferation and biomechanical properties of the murine larynx
Source: PLoS One. 2021 Jan 13;16(1):e0245073. doi: 10.1371/journal.pone.0245073 (PMC7806159; doi:10.1371/journal.pone.0245073)
Supplement: S1 Table — ANOVA reported significance between time points for larynx length (p<0.001), outer dorsoventral diameter (p<0.001), and transverse diameter (p<0.001). A post hoc Tukey test was conducted to report between time point significance. (DOCX) [file pone.0245073.s002.docx]

**S1 Table. P-value results for time point comparisons of larynx growth measurements**.

| Measurement | Time point 1 | Time point 2 | P-value | |
| --- | --- | --- | --- | --- |
| Larynx length | E13.5 | E15.5 | 0.339 |  |
| Larynx length | E13.5 | E16.5 | <0.001* |  |
| Larynx length | E13.5 | E18.5 | <0.001* |  |
| Larynx length | E13.5 | P0 | <0.001* |  |
| Larynx length | E13.5 | Adult | <0.001* |  |
| Larynx length | E15.5 | E16.5 | <0.001* |  |
| Larynx length | E15.5 | E18.5 | <0.001* |  |
| Larynx length | E15.5 | P0 | <0.001* |  |
| Larynx length | E15.5 | Adult | <0.001* |  |
| Larynx length | E16.5 | E18.5 | 0.319 |  |
| Larynx length | E16.5 | P0 | 0.477 |  |
| Larynx length | E16.5 | Adult | <0.001* |  |
| Larynx length | E18.5 | P0 | 1 |  |
| Larynx length | E18.5 | Adult | <0.001* |  |
| Larynx length | P0 | Adult | <0.001* |  |
| Outer dorsoventral diameter | E13.5 | E15.5 | 0.418 |  |
| Outer dorsoventral diameter | E13.5 | E16.5 | <0.001* |  |
| Outer dorsoventral diameter | E13.5 | E18.5 | <0.001* |  |
| Outer dorsoventral diameter | E13.5 | P0 | <0.001* |  |
| Outer dorsoventral diameter | E13.5 | Adult | <0.001* |  |
| Outer dorsoventral diameter | E15.5 | E16.5 | 0.0171* |  |
| Outer dorsoventral diameter | E15.5 | E18.5 | <0.001* |  |
| Outer dorsoventral diameter | E15.5 | P0 | <0.001* |  |
| Outer dorsoventral diameter | E15.5 | Adult | <0.001* |  |
| Outer dorsoventral diameter | E16.5 | E18.5 | 0.024* |  |
| Outer dorsoventral diameter | E16.5 | P0 | <0.001* |  |
| Outer dorsoventral diameter | E16.5 | Adult | <0.001* |  |
| Outer dorsoventral diameter | E18.5 | P0 | 0.147 |  |
| Outer dorsoventral diameter | E18.5 | Adult | <0.001* |  |
| Outer dorsoventral diameter | P0 | Adult | <0.001* |  |
| Transverse diameter | E13.5 | E15.5 | 0.987 |  |
| Transverse diameter | E13.5 | E16.5 | <0.001* |  |
| Transverse diameter | E13.5 | E18.5 | <0.001* |  |
| Transverse diameter | E13.5 | P0 | <0.001* |  |
| Transverse diameter | E13.5 | Adult | <0.001* |  |
| Transverse diameter | E15.5 | E16.5 | <0.001* |  |
| Transverse diameter | E15.5 | E18.5 | <0.001* |  |
| Transverse diameter | E15.5 | P0 | <0.001* |  |
| Transverse diameter | E15.5 | Adult | <0.001* |  |
| Transverse diameter | E16.5 | E18.5 | 0.0141* |  |
| Transverse diameter | E16.5 | P0 | 0.0127* |  |
| Transverse diameter | E16.5 | Adult | <0.001* |  |
| Transverse diameter | E18.5 | P0 | 1 |  |
| Transverse diameter | E18.5 | Adult | <0.001* |  |
| Transverse diameter | P0 | Adult | <0.001* |  |

ANOVA reported significance between time points for larynx length (p<0.001), outer dorsoventral diameter (p<0.001), and transverse diameter (p<0.001). A post hoc Tukey test was conducted to report between time point significance.
