# Supplementary material for: Characterization of intrauterine growth, proliferation and biomechanical properties of the murine larynx
Source: PLoS One. 2021 Jan 13;16(1):e0245073. doi: 10.1371/journal.pone.0245073 (PMC7806159; doi:10.1371/journal.pone.0245073)
Supplement: S2 Table — ANOVA reported significance between time points for VF internal length (p<0.001) and thickness (p<0.001). A post hoc Tukey test was conducted to report between time point significance. (DOCX) [file pone.0245073.s003.docx]

**S2 Table. P-value results for time point comparisons of VF growth measurements**.

| Measurement | Time point 1 | Time point 2 | P-value | |
| --- | --- | --- | --- | --- |
| Internal length | E13.5 | E15.5 | 0.933 |  |
| Internal length | E13.5 | E16.5 | <0.001* |  |
| Internal length | E13.5 | E18.5 | <0.001* |  |
| Internal length | E13.5 | P0 | <0.001* |  |
| Internal length | E13.5 | Adult | <0.001* |  |
| Internal length | E15.5 | E16.5 | <0.001* |  |
| Internal length | E15.5 | E18.5 | <0.001* |  |
| Internal length | E15.5 | P0 | <0.001* |  |
| Internal length | E15.5 | Adult | <0.001* |  |
| Internal length | E16.5 | E18.5 | <0.001* |  |
| Internal length | E16.5 | P0 | <0.001* |  |
| Internal length | E16.5 | Adult | <0.001* |  |
| Internal length | E18.5 | P0 | 1 |  |
| Internal length | E18.5 | Adult | <0.001* |  |
| Internal length | P0 | Adult | <0.001* |  |
| Thickness | E13.5 | E15.5 | <0.001* |  |
| Thickness | E13.5 | E16.5 | <0.001* |  |
| Thickness | E13.5 | E18.5 | <0.001* |  |
| Thickness | E13.5 | P0 | <0.001* |  |
| Thickness | E13.5 | Adult | <0.001* |  |
| Thickness | E15.5 | E16.5 | 0.985 |  |
| Thickness | E15.5 | E18.5 | 0.206 |  |
| Thickness | E15.5 | P0 | 0.0251* |  |
| Thickness | E15.5 | Adult | <0.001* |  |
| Thickness | E16.5 | E18.5 | 0.0389* |  |
| Thickness | E16.5 | P0 | 0.00228* |  |
| Thickness | E16.5 | Adult | <0.001* |  |
| Thickness | E18.5 | P0 | 0.975 |  |
| Thickness | E18.5 | Adult | <0.001* |  |
| Thickness | P0 | Adult | <0.001* |  |

ANOVA reported significance between time points for VF internal length (p<0.001) and thickness (p<0.001). A post hoc Tukey test was conducted to report between time point significance.
