# Supplementary material for: Characterization of intrauterine growth, proliferation and biomechanical properties of the murine larynx
Source: PLoS One. 2021 Jan 13;16(1):e0245073. doi: 10.1371/journal.pone.0245073 (PMC7806159; doi:10.1371/journal.pone.0245073)
Supplement: S3 Table — ANOVA reported significance between time points for coronal (p<0.001) and transverse (p<0.001) proliferation. A post hoc Tukey test was conducted to report between time point significance. (DOCX) [file pone.0245073.s004.docx]

**S3 Table. P-value results for time point comparisons of proliferation measurements**.

| Measurement | Time point 1 | Time point 2 | P-value | |
| --- | --- | --- | --- | --- |
| Coronal proliferation | E13.5 | E15.5 | <0.001* |  |
| Coronal proliferation | E13.5 | E16.5 | <0.001* |  |
| Coronal proliferation | E13.5 | E18.5 | <0.001* |  |
| Coronal proliferation | E13.5 | P0 | <0.001* |  |
| Coronal proliferation | E13.5 | Adult | <0.001* |  |
| Coronal proliferation | E15.5 | E16.5 | 0.846 |  |
| Coronal proliferation | E15.5 | E18.5 | 0.257 |  |
| Coronal proliferation | E15.5 | P0 | 0.846 |  |
| Coronal proliferation | E15.5 | Adult | 0.144 |  |
| Coronal proliferation | E16.5 | E18.5 | 0.846 |  |
| Coronal proliferation | E16.5 | P0 | 1 |  |
| Coronal proliferation | E16.5 | Adult | 0.643 |  |
| Coronal proliferation | E18.5 | P0 | 0.846 |  |
| Coronal proliferation | E18.5 | Adult | 0.999 |  |
| Coronal proliferation | P0 | Adult | 0.643 |  |
| Transverse proliferation | E13.5 | E15.5 | 0.0128* |  |
| Transverse proliferation | E13.5 | E16.5 | <0.001* |  |
| Transverse proliferation | E13.5 | E18.5 | <0.001* |  |
| Transverse proliferation | E13.5 | P0 | <0.001* |  |
| Transverse proliferation | E13.5 | Adult | <0.001* |  |
| Transverse proliferation | E15.5 | E16.5 | 0.0128* |  |
| Transverse proliferation | E15.5 | E18.5 | 0.028* |  |
| Transverse proliferation | E15.5 | P0 | 0.0189* |  |
| Transverse proliferation | E15.5 | Adult | 0.00406* |  |
| Transverse proliferation | E16.5 | E18.5 | 0.997 |  |
| Transverse proliferation | E16.5 | P0 | 1 |  |
| Transverse proliferation | E16.5 | Adult | 0.979 |  |
| Transverse proliferation | E18.5 | P0 | 1 |  |
| Transverse proliferation | E18.5 | Adult | 0.848 |  |
| Transverse proliferation | P0 | Adult | 0.932 |  |

ANOVA reported significance between time points for coronal (p<0.001) and transverse (p<0.001) proliferation. A post hoc Tukey test was conducted to report between time point significance.
