# Supplementary material for: Characterization of intrauterine growth, proliferation and biomechanical properties of the murine larynx
Source: PLoS One. 2021 Jan 13;16(1):e0245073. doi: 10.1371/journal.pone.0245073 (PMC7806159; doi:10.1371/journal.pone.0245073)
Supplement: S4 Table — Welch’s ANOVA reported significance between time points for stiffness (p<0.001). A post hoc Games-Howell test was conducted to report between time point significance. (DOCX) [file pone.0245073.s005.docx]

**S4 Table. P-value results for time point comparisons of stiffness measurements**.

| Measurement | Time point 1 | Time point 2 | P-value | |
| --- | --- | --- | --- | --- |
| Stiffness | E13.5 | E15.5 | 0.75 |  |
| Stiffness | E13.5 | E16.5 | <0.001* |  |
| Stiffness | E13.5 | E18.5 | <0.001* |  |
| Stiffness | E13.5 | P0 | <0.001* |  |
| Stiffness | E13.5 | Adult | <0.001* |  |
| Stiffness | E15.5 | E16.5 | <0.001* |  |
| Stiffness | E15.5 | E18.5 | <0.001* |  |
| Stiffness | E15.5 | P0 | <0.001* |  |
| Stiffness | E15.5 | Adult | <0.001* |  |
| Stiffness | E16.5 | E18.5 | <0.001* |  |
| Stiffness | E16.5 | P0 | <0.001* |  |
| Stiffness | E16.5 | Adult | <0.001* |  |
| Stiffness | E18.5 | P0 | <0.001* |  |
| Stiffness | E18.5 | Adult | <0.001* |  |
| Stiffness | P0 | Adult | <0.001* |  |

Welch’s ANOVA reported significance between time points for stiffness (p<0.001). A post hoc Games-Howell test was conducted to report between time point significance.
